# Age-specific SARS-CoV-2 infection fatality ratio and associated risk factors, Italy, February to April 2020

**DOI:** 10.2807/1560-7917.ES.2020.25.31.2001383

**Published:** 2020-08-06

**Authors:** Piero Poletti, Marcello Tirani, Danilo Cereda, Filippo Trentini, Giorgio Guzzetta, Valentina Marziano, Sabrina Buoro, Simona Riboli, Lucia Crottogini, Raffaella Piccarreta, Alessandra Piatti, Giacomo Grasselli, Alessia Melegaro, Maria Gramegna, Marco Ajelli, Stefano Merler

**Affiliations:** 1 Bruno Kessler Foundation, Trento, Italy; 2 Directorate General for Health, Lombardy Region, Milan, Italy; 3 Health Protection Agency of Pavia, Pavia, Italy; 4 Quality Management Unit, Papa Giovanni XXIII Hospital, Bergamo, Italy; 5 Department of Public Health, Experimental and Forensic Medicine, University of Pavia, Pavia, Italy; 6 Dondena Centre for Research on Social Dynamics and Public Policy, Bocconi University, Milan, Italy; 7 Department of Decision Sciences, Bocconi University, Milan, Italy; 8 Dipartimento di Anestesia, Rianimazione ed Emergenza-Urgenza, Fondazione IRCCS Ca’ Granda Ospedale Maggiore Policlinico, Milan, Italy; 9 Dipartimento di Fisiopatologia Medico-Chirurgica e dei Trapianti, Università degli Studi di Milano, Milan, Italy; 10 Department of Social and Political Sciences, Bocconi University, Milan, Italy; 11 Department of Epidemiology and Biostatistics, Indiana University School of Public Health, Bloomington, United States

**Keywords:** SARS-CoV-2, COVID-19, infection fatality ratio, mortality, comorbidities

## Abstract

We analysed 5,484 close contacts of coronavirus disease (COVID-19) cases in Italy, all tested for severe acute respiratory syndrome coronavirus 2 (SARS-CoV-2). Infection fatality ratio was 0.43% (95% confidence interval (CI): 0.21–0.79) for individuals younger than 70 years and 10.5% (95% CI: 8.0–13.6) for older individuals. Risk of death after infection was 62% lower (95% CI: 31–80) in clusters identified after 16 March 2020 and 1.8-fold higher for males (95% CI: 1.03–3.16).

Coronavirus disease (COVID-19) still represents a major global health threat as the majority of the world’s population remains susceptible to severe acute respiratory syndrome coronavirus 2 (SARS-CoV-2) [1-3]. The proportion of infections resulting in a fatal outcome, known as infection fatality ratio (IFR), and the associated risk factors are still poorly quantified for SARS-CoV-2 [1,4].

The aim of this study was to provide estimates of SARS-CoV-2 IFR stratified by age, sex and comorbidities and to investigate the risk factors for fatal outcome in SARS-CoV-2-infected people.

## Study population and sample description

The study population comprised contacts of COVID-19 cases identified through contact tracing conducted in Lombardy, Italy between February and April 2020. For these subjects, we analysed reverse-transcription PCR (RT-PCR) results from nasopharyngeal swabs administered during the contact tracing activity. These data were complemented with the results of an ongoing serological survey on the same group of individuals that started on 16 April 2020. Finally, we also collected information on comorbidities (respiratory, cardiovascular, metabolic and oncological) and clinical outcomes of each case reported in the Lombardy linelist of COVID-19 patients (last update: 8 June 2020).

The data analysed here represent a selection from a database of 62,881 contacts of COVID-19 cases. We selected only contacts belonging to clusters (i.e. groups of contacts identified by one positive index case) where all individuals were tested against SARS-CoV-2 infection either through nasal swabs during the contact tracing operations or through retrospective IgG serological testing. To avoid possible biases, we excluded the index cases as they were often identified because of their symptoms and may therefore have been at higher risk of severe disease. The accuracy of IgG testing and RT-PCR used in our sample was assessed in [5,6]. The IFR was computed as the proportion of deaths that occurred among all SARS-CoV-2-positive contacts identified in the considered sample, here defined as subjects with at least one laboratory confirmation of their infection.

Overall, we analysed 5,484 contacts (median age: 50 years; IQR: 30–61; 43.7% male). Of those, 1,364 (25%) were tested only by an RT-PCR assay targeting different SARS-CoV-2 genes during the contact tracing activities [5], 3,493 (64%) were tested only by a serological assay for IgG neutralising antibodies against S1/S2 antigens [6] at least a month after the reporting date of their index case and 627 (11%) were tested both by RT-PCR and serology. Among the 5,484 analysed individuals, 2,824 resulted positive to SARS-CoV-2 (median age: 53 years; IQR: 34–64; 43.2% male) and 62 of the positive individuals died with a COVID-19 diagnosis (median age: 79 years; IQR: 74–83; 53.2% male).

## Estimates of the infection fatality ratio stratified by age, sex, and comorbidities

We performed a univariate analysis and estimated the mean IFR at 10.5% (95% confidence interval (CI): 8.0–13.6) for individuals 70 years and older and at 0.43% (95% CI: 0.21–0.79) for those younger than 70 years (Table 1 and Figure). No deaths were recorded among individuals younger than 50 years. The IFR was higher in men than in women: 14.0% (95% CI:  9.4–19.7) vs 8.3% (95% CI: 5.5–12.1) in subjects 70 years and older and 0.58% (95% CI: 0.21–1.27%) vs 0.31% (95% CI: 0.08–0.78) in younger subjects. The IFR was remarkably lower for patients associated with clusters identified after 16 March 2020 (the median date of confirmation among index cases in the considered clusters), especially among infections in people 80 years and older: 3.70% (95% CI: 0.45–12.8) vs 28.2% (95% CI: 15.0–44.9) in women and 15.6% (95% CI: 5.28–32.8) vs 33.3% (95% CI: 17.3–52.8) in men. In our sample, 51 of 62 deaths occurred in patients affected by cardiovascular diseases (which include hypertension, hypercholesterolemia, myocardiopathy, heart failure, ischemic and valve cardiopathy, arterial and venous vasculopathy). The IFR for subjects with this comorbidity was 22.44% (95% CI: 16.15–29.80) before 16 March 2020 and 9.68% (95% CI: 5.52–15.46) after. The sample size was too small for a useful multivariate analysis.

**Table 1:** Sample description and infection fatality ratio estimates by sex, age group and comorbidities, close contacts of COVID-19 cases, Lombardy, Italy, February–April 2020 (n = 5,484)

	Any time (n = 5,484)				Before 16 March 2020 (n = 2,696**^a^**)				After 16 March 2020 (n = 2,721**^a^**)			
	Contacts	SARS-CoV-2-positive	Deaths	Mean IFR in % (95% CI)	Contacts	SARS-CoV-2-positive	Deaths	Mean IFR in % (95% CI)	Contacts	SARS-CoV-2-positive	Deaths	Mean IFR in % (95% CI)
**Age (years)**												
0–19	692	304	0	0 (0–1.21)	273	114	0	0 (0–3.18)	413	188	0	0 (0–1.94)
20–49	1,951	885	0	0 (0–0.42)	951	438	0	0 (0–0.84)	973	431	0	0 (0–0.85)
50–59	1,241	648	3	0.46 (0.1–1.35)	663	354	2	0.56 (0.07–2.03)	560	283	1	0.35 (0.01–1.95)
60–69	867	494	7	1.42 (0.57–2.9)	448	259	4	1.54 (0.42–3.91)	409	227	2	0.88 (0.11–3.15)
70–79	485	335	23	6.87 (4.4–10.12)	253	189	15	7.94 (4.51–12.75)	229	143	8	5.59 (2.45–10.73)
≥ 80	248	158	29	18.35 (12.65–25.28)	108	69	21	30.43 (19.92–42.69)	137	86	7	8.14 (3.34–16.05)
**Sex^b^**												
Male	2,398	1,220	33	2.7 (1.87–3.78)	1,114	587	23	3.92 (2.5–5.82)	1,254	615	9	1.46 (0.67–2.76)
Female	3,086	1,604	29	1.81 (1.21–2.59)	1,582	836	19	2.27 (1.37–3.53)	1467	743	9	1.21 (0.56–2.29)
**Comorbidities^b^**												
None	122	113	1	0.88 (0.02–4.83)	53	49	0	0 (0–7.25)	69	64	1	1.56 (0.04–8.4)
Cardiovascular^c^	350	316	51	16.14 (12.26–20.67)	173	156	35	22.44 (16.15–29.8)	172	155	15	9.68 (5.52–15.46)
Respiratory^c^	50	49	8	16.33 (7.32–29.66)	23	23	6	26.09 (10.23–48.41)	24	24	2	8.33 (1.03–27)
Oncological^c^	106	92	11	11.96 (6.12–20.39)	55	51	6	11.76 (4.44–23.87)	47	38	4	10.53 (2.94–24.8)
Diabetes/metabolic^c^	93	79	13	16.46 (9.06–26.49)	43	37	11	29.73 (15.87–46.98)	48	40	2	5 (0.61–16.92)
Unknown	4,947	2,335	9	0.39 (0.18–0.73)	2,437	1,186	6	0.51 (0.19–1.1)	2,452	1,114	2	0.18 (0.02–0.65)

CI: confidence interval; IFR: infection fatality ratio; SARS-CoV-2: severe acute respiratory syndrome coronavirus 2.

^a^ Information on the epidemic period was not available for 67 close case contacts, including 43 SARS-CoV-2 infections and two deaths.

^b^ Estimates obtained by aggregating the sample irrespective of age should be cautiously interpreted as the sample may not reflect age-specific SARS-CoV-2 immunity in the population.

^c^ Patients with more than one comorbidity are counted multiple times.

**Figure:**
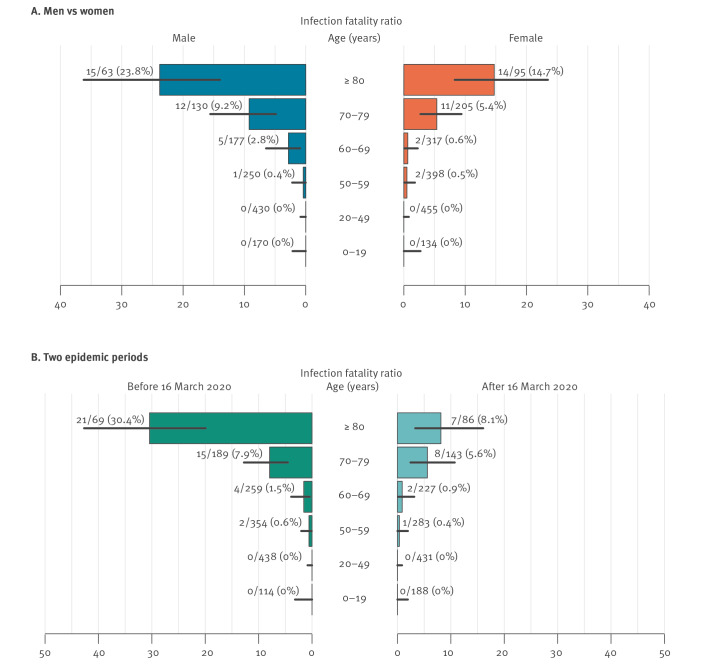
Age-specific estimates (mean) of infection fatality ratio, SARS-CoV-2-positive contacts, Lombardy, Italy, February–April 2020 (n = 2,824)

Horizontal lines represent 95% confidence intervals computed by exact binomial tests.

## Risk factors associated with fatal outcome

To identify the risk factors associated with fatal outcome after SARS-CoV-2 infection, we applied a generalised linear model (GLM with logit link) relating the observed outcome (death vs survival) to the sex and the age group (0–59, 60–69, 70–79, ≥ 80 years) of the exposed individuals, to the presence of comorbidities (none, cardiovascular, others), and to the epidemic period of the observed outcomes (before or after 16 March 2020).

We found that individuals younger than 70 years were at a significantly lower risk of death after infection than older patients (Tukey test: p value < 0.001). The relative risk (RR) of death was 1.81 higher in men than in women (95% CI: 1.03–3.16; Tukey test: p value < 0.001) and 5.6 times higher for patients affected by cardiovascular comorbidities (95% CI: 1.2–55.3) compared with otherwise healthy individuals, although the latter difference was not statistically significant (Tukey test: p value = 0.29). Finally, the risk of death was 62% lower (95% CI: 31–80%; Tukey test: p value < 0.001) during the second phase of the epidemic (Table 2).

**Table 2:** Estimated relative risk of death after SARS-CoV-2 infection, Lombardy, Italy, SARS-CoV-2-positive contacts, Lombardy, Italy, February–April 2020 (n = 2,824)

	SARS-CoV-2-positive	Deaths	RR (95% CI)
**Age (years)**			
≥ 80	158	29	Reference
0–59	1,837	3	0.03 (0.01–0.1)
60–69	494	7	0.14 (0.05–0.32)
70–79	335	23	0.5 (0.27–0.89)
**Sex**			
Female	1,604	29	Reference
Male	1,220	33	1.81 (1.03–3.16)
**Comorbidity**			
None	113	1	Reference
Cardiovascular	316	51	5.64 (1.17–55.27)
Other comorbidity	60	1	0.93 (0.04–20.55)
Unknown	2,335	9	0.36 (0.06–6.42)
**Epidemic period**			
Before 16 March 2020	1,423	42	Reference
After 16 March 2020	1,358	18	0.38 (0.2–0.69)
Unknown	43	2	3.09 (0.43–10.94)

CI: confidence interval; RR: relative risk of death after infection; SARS-CoV-2: severe acute respiratory syndrome coronavirus 2.

We used a generalised linear model where the fatal outcome is used as the response variable and sex, age group, comorbidities and epidemic period are considered as regressors.

We also performed regression analyses by adding interaction terms to the specifications used in the main analysis. These models were inferior in terms of likelihood ratios ruling out the possible nonlinearities. We also considered a model explicitly accounting for the number of comorbidities but did not observed a statistically significant correlation.

## Discussion

Estimates of the IFR are key to evaluating the health impact of epidemics and the effectiveness of control strategies [2,7]. Given the high proportion of asymptomatic and pauci-symptomatic SARS-CoV-2 infections [8,9], it is difficult to estimate the IFR from surveillance data [1,2,7]. In addition, the proportion of ascertained cases over all infections can change dramatically across regions and over time [2,7]. The increasing availability of serological data can assist precise and direct measurements of the IFR. Literature estimates available so far are highly variable (ranging from 0.07% to 1.6%) [1,2,4,10,11], based on small non-random samples [9], data pooled from heterogeneous populations [1,2,4,10] or derived through modelling analyses [2].

Our age-specific estimates of the IFR compare well with results from previous studies in China [2] and Switzerland [12]. More than 80% of the deaths recorded in our sample occurred in patients with cardiovascular diseases, the most common comorbidity among patients hospitalised in the early phase of the Italian epidemic [3]. Owing to the limited sample size we are unable to provide solid estimates of the IFR stratified by both age and comorbidities. However, our results suggest that the higher number of COVID-19 deaths reported in Italy compared with other countries may be due to the high proportion of older individuals in the Lombardy population (28.7% of people older than 60 years vs the European average 25.7%) or to differences in the prevalence of chronic diseases. Our estimates should also be interpreted in light of the specific area and time period considered here, mirroring a health system under severe strain because of the rapid increase in patients requiring intensive care [3]. These conditions combined with the initial scant evidence on appropriate treatments may well have affected the health system’s capacity to cope with severe cases. This hypothesis is supported by the remarkable difference in the IFR estimates for clusters identified before and after 16 March.

The age distribution in our sample reflects that of the Lombardy population (Supplementary Figure S1). However, the data considered in this study represent a sample of individuals who were exposed to COVID-19 cases. Therefore, the reported infection attack rates cannot be considered as representative for the entire region. This also means that estimates of the overall IFR that could be computed by aggregating the entire sample irrespective of age (namely: 2.2%), although compliant with the range of IFR obtained for Spain (between 1% and 2% [1]), should be interpreted with caution. Similar arguments apply for the estimated IFR in men and women when not disaggregated by age.

A limitation of this analysis is that infections were identified in two different periods of time and using different tests (RT-PCR and IgG serological assays). In our sample, all contacts were followed to detect possible symptoms in the time interval form the exposure to the end of the observation period, but while symptomatic individuals were tested by PCR shortly after their index case was identified, IgG serological tests were performed on individuals more than 1 month after their identification as contacts. These features minimise potential biases related to identification of infections and deaths or to seroconversion delays. However, of 327 contacts tested by both RT-PCR and serology, 137 resulted negative in RT-PCR and positive in serology (Supplementary Table S1). We explored to what extent failures in RT-PCR testing may have affected estimates of the overall IFR, by considering a worst-case scenario where 41.9% (i.e. 137/327) of negative contacts who were tested with RT-PCR only (n = 732) were assumed to be positive. The estimated IFR in this case becomes ca 8% smaller than the one obtained with the baseline analysis (12% and 4% before and after 16 March 2020, respectively; for details see the Supplement). We explored to what extent false-positive results arising from IgG testing (specificity: 98.3% [11]) may have impacted estimates on the overall IFR, by considering a worst-case scenario where 1.7% of the 1,892 positive contacts who were tested with IgG and were not confirmed by positive RT-PCR results were assumed to be negative. The differences to the baseline estimates were negligible (Supplement).

## Conclusion

The estimates provided here can be considered a robust representation of the IFR during the COVID-19 epidemic in Lombardy, the most affected Italian region [3,5]. These results can be instrumental in evaluating the expected burden of possible future outbreaks. The indication on the key factors that strongly influence the SARS-CoV-2 IFR could be used to inform targeted interventions and possible future COVID-19 vaccination campaigns.

## Supplementary Data

144000 Supplement

## References

[1] Okell LC Verity R Watson OJ Mishra S Walker P Whittaker C Have deaths from COVID-19 in Europe plateaued due to herd immunity? Lancet. 2020;395(10241):e110-1. 10.1016/S0140-6736(20)31357-X 32534627 PMC7289569

[2] Verity R Okell LC Dorigatti I Winskill P Whittaker C Imai N Estimates of the severity of coronavirus disease 2019: a model-based analysis. Lancet Infect Dis. 2020;20(6):669-77. 10.1016/S1473-3099(20)30243-7 32240634 PMC7158570

[3] Grasselli G Zangrillo A Zanella A Antonelli M Cabrini L Castelli A Baseline characteristics and outcomes of 1591 patients infected with SARS-CoV-2 admitted to ICUs of the Lombardy region, Italy. JAMA. 2020;323(16):1574-81. 10.1001/jama.2020.5394 32250385 PMC7136855

[4] Meyerowitz-Katz G and Merone L (2020) A systematic review and meta-analysis of published research data on COVID-19 infection-fatality rates. medRxiv. 2020. Available from: https://doi.org/10.1101/2020.05.03.20089854 10.1016/j.ijid.2020.09.1464 PMC7524446 33007452

[5] Cereda D, Tirani M, Rovida F, Demicheli V, Ajelli M, Poletti P, et al. The early phase of the COVID-19 outbreak in Lombardy, Italy. arXiv. 2020. Available from: https://arxiv.org/abs/2003.09320

[6] Bonelli F Sarasini A Zierold C Calleri M Bonetti A Vismara C Clinical and analytical performance of an automated serological test that identifies S1/S2 neutralizing IgG In Covid-19 patients semiquantitatively. J Clin Microbiol. 2020;JCM.01224-20:JCM.01224-20. 10.1128/JCM.01224-20 32580948 PMC7448652

[7] Rajgor DD Lee MH Archuleta S Bagdasarian N Quek SC The many estimates of the COVID-19 case fatality rate. Lancet Infect Dis. 2020;20(7):776-7. 10.1016/S1473-3099(20)30244-9 32224313 PMC7270047

[8] Poletti P, Tirani M, Cereda D, Trentini F, Guzzetta G, Sabatino G, et al. Probability of symptoms and critical disease after SARS-CoV-2 infection. arXiv. 2020. Available from: https://arxiv.org/abs/2006.08471

[9] Buitrago-Garcia DC, Egli-Gany D, Counotte MJ, Hossmann S, Imeri H, Salanti G, et al. The role of asymptomatic SARS-CoV-2 infections: rapid living systematic review and meta-analysis. medRxiv. 2020. Available from: https://doi.org/10.1101/2020.04.25.20079103 10.1371/journal.pmed.1003346 PMC7508369 32960881

[10] Russell TW Hellewell J Jarvis CI van Zandvoort K Abbott S Ratnayake R Estimating the infection and case fatality ratio for coronavirus disease (COVID-19) using age-adjusted data from the outbreak on the Diamond Princess cruise ship, February 2020. Euro Surveill. 2020;25(12):2000256. 10.2807/1560-7917.ES.2020.25.12.2000256 32234121 PMC7118348

[11] Erikstrup C, Hother CE, Pedersen OBV, Mølbak K, Skov RL, Holm DK, et al. Estimation of SARS-CoV-2 infection fatality rate by real-time antibody screening of blood donors. medRxiv. 2020. Available from: https://doi.org/10.1101/2020.04.24.20075291 10.1093/cid/ciaa849 PMC7337681 33501969

[12] Perez-Saez J Lauer SA Kaiser L Regard S Delaporte E Guessous I Serology-informed estimates of SARS-CoV-2 infection fatality risk in Geneva, Switzerland. Lancet Infect Dis. 2020;S1473-3099(20)30584-3. 10.1016/S1473-3099(20)30584-3 32679085 PMC7833057

