# Supplementary material for: Age-specific SARS-CoV-2 infection fatality ratio and associated risk factors, Italy, February to April 2020
Source: Euro Surveill. 2020 Aug 6;25(31):2001383. doi: 10.2807/1560-7917.ES.2020.25.31.2001383 (PMC7459272; doi:10.2807/1560-7917.ES.2020.25.31.2001383)
Supplement: Supplement [file 20-01383_POLETTI_Supplement.pdf]

Supplement S1:

Age-specific SARS-CoV-2 infection fatality ratio and associated risk factors, Italy, February to April 2020

Piero Poletti, Marcello Tirani, Danilo Cereda, Filippo Trentini, Giorgio Guzzetta, Valentina Marziano, Sabrina Buoro, Simona Riboli, Lucia Crottogini, Raffaella Piccarreta, Alessandra Piatti, Giacomo Grasselli, Alessia Melegaro, Maria Gramegna, Marco Ajelli, Stefano Merler

Disclaimer: This supplementary material is hosted by Eurosurveillance as supporting information alongside the article "Age-specific SARS-CoV-2 infection fatality ratio and associated risk factors, Italy, February to April 2020", on behalf of the authors, who remain responsible for the accuracy and appropriateness of the content. The same standards for ethics, copyright, attributions and permissions as for the article apply. Supplements are not edited by Eurosurveillance and the journal is not responsible for the maintenance of any links or email addresses provided therein.

Materials and Methods

Data

The data analyzed in this study were taken from a database providing, among other details, information on sex, age, presence of comorbidities for close case contacts, the results of RT-PCR tests and serological tests (if any), and the clinical outcome of positive cases. In particular, the database was built by combining:
i) data records collected during the contact tracing activities conducted between February 21 and April 16, 2020 by the Lombardy healthcare agencies;
ii) results of RT PCR assays on nasopharyngeal swabs mainly administered to symptomatic contacts short after their identification during contact tracing;
iii) results from serological assays collected within a seroprevalence study started on April 16, 2020 and not yet completed, which mainly targets case contacts who were not tested by RT-PCR or resulted negative to RT-PCR;
iv) information (updated to June 8, 2020) on the clinical outcome of patients as available in the linelist of all COVID-19 laboratory confirmed cases in Lombardy.

Contact data collected after April 16, 2020 were excluded to avoid biases caused by delays in development of symptoms, reporting, and in seroconversion of infected individuals. The performed analysis is based on the serological test results obtained by May 25, 2020 and on the linelist of all COVID-19 cases detected in the region, as updated on June 8, 2020. In this study, we selected only contacts belonging to clusters with complete testing i.e. clusters whose contacts all received at least one between a valid RT-PCR result or a valid serological result [1]. Clusters with case contacts with inconclusive serological results were excluded by the proposed analysis. Specifically, out of the 21,519 clusters identified in Lombardy before April 16, 2020, 90 (0.42%) were excluded due to 101 contacts (0.16% of all case contacts) with inconclusive serological results; 18,009 clusters (83.7%) were excluded due to incomplete testing.

A close contact of a case was defined as either of the following:
- a person living in the same household as a COVID-19 confirmed case;
- a person having had face-to-face interaction with a COVID-19 confirmed case;
- a person who was in a closed environment (e.g. classroom, meeting room, hospital waiting room) with a COVID-19 confirmed case at a distance of less than 2 meters for more than 15 minutes;
- a healthcare worker or other person providing direct care for a COVID-19 confirmed case, or laboratory workers handling specimens from a COVID-19 confirmed case without recommended personal protective equipment (PPE) or with a possible breach of PPE;

- a contact in an aircraft sitting within two seats (in any direction) of a COVID-19 confirmed case, travel companions or persons providing care, and crew members serving in the section of the aircraft where the index case was seated; passengers seated in the entire section or all passengers on the aircraft were considered close contacts of a confirmed case when severity of symptoms or movement of the case indicated more extensive exposure.

Confirmed cases were defined as subjects with positive laboratory confirmation via RT-PCR assays for virus causing of SARS-CoV-2 infection, irrespective of clinical signs and symptoms.

A cluster of contacts was defined as the set of contacts identified by one positive index case. Contact tracing activities were carried out through standardized epidemiological investigations of positive cases (or of their parents/relatives) to determine the history of individuals' exposure. The exposure period was initially defined as the time interval ranging from 14 days before to 14 days after the symptom onset of an index case. Following guidelines from WHO [2], after March 20 the time interval was changed from 2 days before to 14 days after the symptom onset of the index case.

Confirmation of cases was obtained with nasal swabs (UTM viral transport ®, Copan Italia S.p.a) with at least two real-time RT-PCR assays targeting different genes (E and RdRp) of SARS-CoV-2 [3,4]. In addition, a novel quantitative real-time RT-PCR targeting an additional SARS-CoV-2 gene (M) was developed (details provided upon request). From February 21 to February 25, all contacts were tested for the presence of viral genome on their nasopharyngeal trait. To economize the saturated testing resources in Lombardy, from February 26, 2020 onward (i.e., shortly after the detection of the first locally transmitted COVID-19 case in Italy) only symptomatic contacts of COVID-19 cases were tested via RT-PCR. From March 20, positivity of nasal swabs was also ascertained from a test that sought a single gene. Individuals with inconclusive tests were swabbed again and re-tested to resolve the diagnosis.

On April 16, 2020, the regional health authorities started a serological survey aiming at completing the ascertainment of SARS-CoV-2 infections among all close contacts identified for each confirmed case. Serological screening included both symptomatic and asymptomatic case contacts identified through contact tracing activities without history of a swab for SARS-CoV-2. A relatively small number of serological tests were administered to individuals already tested by RT-PCR (n=627 in our sample). The test used to detect SARS-CoV-2 IgG antibodies is the LIAISON® SARS-CoV-2 test [5,6]. The LIAISON® SARS-CoV-2 test employs magnetic beads coated with S1 & S2 antigens. The antigens used in the tests are expressed in human cells to achieve proper folding, oligomer formation, and glycosylation, providing material similar to the native spikes. This strategy ensures that the antigen-antibody complex forms with the required specificity. The S1 and S2 proteins are both targets to neutralizing antibodies. The test provides the detection of IgG antibodies against S1/S2 antigens of SARS-CoV-2 and the detection of neutralizing antibodies with 98.3% specificity and 94.4% sensitivity at 15 days from diagnosis [5,6] and validated against Plaque Reduction Neutralization Test (PRNT) [5]. A negative result (<12 AU/mL) indicates the absence or a very low level of IgG antibodies directed against the virus; this occurs in the absence of infection or during the incubation period or in the early stages of the disease. An inconclusive result (12-15 AU/mL) can be interpreted as both a false positive or a false negative and suggests repeating the exam after a week. A positive result (>15 AU/mL) indicates the presence of IgG antibodies and therefore a past infection with SARS-CoV-2.

Statistical analysis

Contact tracing data combined with test results and outcomes of close contacts associated with each index case were used to estimate the SARS-CoV-2 infection fatality rate (IFR). The IFR was estimated for male and female individuals separately and for five age groups (0-19 years, 20-49 years, 50-59 years, 60-69 years, 70-79 years, 80+ years) in two distinct epidemic phases (before and after March 16, 2020) and for patients affected by cardiovascular comorbidities (including hypertension) and patients with no comorbidities. The IFR was computed as the proportion of deaths among the total number of infected individuals for each considered strata. Exact binomial test was used to estimate confidence intervals.

To investigate IFR risk factors, we used a generalized linear model (GLM with logit link), using the outcome of positive close contacts as a binary response variable (i.e. death vs. survival) and considering the following covariates:

- the age group of the contact; as no deaths were found below 50 years of age, four age groups were considered in this case: 0-59 years, 60-69 years, 70-79 years, 80+ years.
- the sex of the contact;
- a categorical variable defining whether: 1) the contact was presenting at least one cardiovascular comorbidity, including hypertension; 2) the contact was not presenting cardiovascular comorbidities but was presenting at least one of the following comorbidities: respiratory, oncological, metabolic (including diabetes); 3) no comorbidities were known for the contact;
- a binary variable defining whether the date of identification of the index case associated to the contact was prior or posterior to March 16, 2020; this date represents the median date of confirmation among index cases in the selected clusters (i.e. those with complete testing).

Regression models including interactions between covariates or considering the number of comorbidities affecting a contact were ruled out when compared to model described above on the basis of likelihood ratio tests. Regression models using a numeric variable for the number of comorbidities or a binary variable for the presence of each type of comorbidity instead of the above described categorical variable were ruled out on the basis of the Akaike information criterion.

Risk ratios of death after infection were computed given the covariates, using the generalized linear model described above. Resulting means were compared by Tukey post-hoc test. The statistical analysis was performed using R (version 3.6).

Finally, in the analyzed sample (Table S1), 41.9% (137/327) contacts tested by both RT-PCR and serology resulted negative to RT-PCR and positive to serology. This result may be due to a variety of factors including false positive results from RT-PCR, false negative results from serology, late PCR testing of SARS-CoV-2 infections, transmission occurred outside the analyzed clusters. Although our data are not appropriate to evaluate the accuracy of PCR and serological tests, we performed a sensitivity analysis to explore to what extent false negative PCR results can affect our estimates on the IFR. To do this, we computed the overall IFR resulting form 10,000 simulations where, a random sample of 41.9% contacts who were RT-PCR negative contacts and were not serologically tested (namely 732) is assumed to be positive to SARS-CoV-2. In this case the IFR results 2.04% (95%CI 1.57-2.60%) instead of the 2.2% (95%CI 1.69-2.81%), obtained when pooling all records used in our baseline analysis (i.e. computing the IFR irrespectively to our sample representativeness of the age-specific infection attack rates in the general population). When stratified for the two epidemic periods, the overall IFR obtained in this sensitivity was 2.64% (95%CI 1.91-3.55) for the period before March 16 (as compared to 2.95%, 95%CI 2.14-3.97 reported in the main analysis) and 1.27% (95%CI 0.77-1.98) for the second period (as compared to 1.33%, 95%CI 0.79-2.09 reported in the main analysis).

Following a similar approach, we conducted a second sensitivity analysis to explore to what extent false positive arising from IgG testing may impact estimates on the overall IFR. Given the 98.3% specificity of the IgG test used in our sample, we computed the overall IFR resulting form 10,000 simulations where, a random sample of 1.7% contacts positive to IgG that were not confirmed by RT-PCR results (namely 1,892) is assumed to be negative to SARS-CoV-2. In this case, the mean of the overall IFR was estimted to be 2.22% (95%CI 1.71-2.84), as compared to 2.20% (95%CI 1.69-2.81%) reported in the main analysis. The IFRs stratified for the first and second epidemic period were 2.98% (95%CI 2.16-4.01) and 1.34% (95%CI 0.8-2.11), respectively.

Supplementary tables and figures

RT-PCR	Serological assay (IgG)	Total
Performed	Not performed	1,364
Positive	-	632
Not performed	Performed	3,493
-	Positive	1,755
Performed	Performed	627
Positive	Negative	5
Negative	Positive	137
Positive	Positive	295
Negative	Negative	190

Table S1. Sample description. In Lombardy, from February 26 onward, only symptomatic contacts of COVID-19 confirmed cases were tested with RT-PCR. Out of 1,991 RT-PCR tests, 1,947 were conducted after February 26.

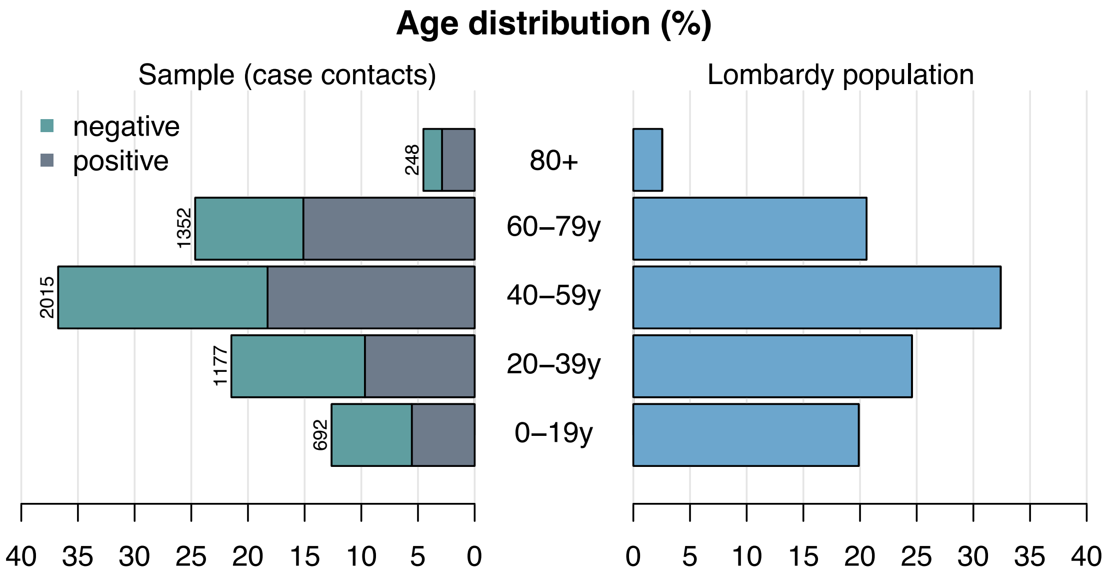

Figure S1. Comparison of the age distribution of analyzed close contacts (darker bars represent SARS-CoV-2 positive individuals) with the age distribution of the Lombardy population in 2019, as reported by the Italian National Institute of Statistics [7]

References

[1] Poletti P, et al (2020) Probability of symptoms and critical disease after SARS-CoV-2 infection. Arxiv https://arxiv.org/abs/2006.08471

[2] World Health Organization. (2020). Contact tracing in the context of COVID-19: interim guidance, 10 May 2020. Available from: https://apps.who.int/iris/handle/10665/332049

[3] Cereda D, et al. (2020) The early phase of the COVID-19 outbreak in Lombardy, Italy. 23 Mar 2020. Available from: https://arxiv.org/abs/2003.09320

[4] Corman VM, et al. (2020) Detection of 2019 novel coronavirus (2019-nCoV) by real-time RT-PCR. Euro Surveill

[5] Bonelli F, et al (2020) Clinical and analytical performance of an automated serological test that identifies S1/S2 neutralizing IgG in Covid-19 patients semiquantitatively. biorxiv https://www.biorxiv.org/content/10.1101/2020.05.19.105445v1.abstract

[6] Geurts van Kessel C, et al (2020). Towards the next phase: evaluation of serological assays for diagnostics and exposure assessment. medrxiv https://www.medrxiv.org/content/10.1101/2020.04.23.20077156v2.full.pdf

[7] Italian National Institute of Statistics. Popolazione residente per età, sesso e stato civile al 1° Gennaio 2019. Accessed on March 1st, 2020. Available from: http://demo.istat.it/pop2019/index.html.
